# Supplementary material for: The interactome of intact mitochondria by cross-linking mass spectrometry provides evidence for coexisting respiratory supercomplexes
Source: Mol Cell Proteomics. 2017 Dec 8;17(2):216–32. doi: 10.1074/mcp.RA117.000470 (PMC5795388; doi:10.1074/mcp.RA117.000470)
Supplement: Supplemental Data [file supp_RA117.000470_133922_0_supp_23978_fzffwf.zip › spectra_annotation/mito_spectra_annotation/316-2-6-1-6-1.pdf]

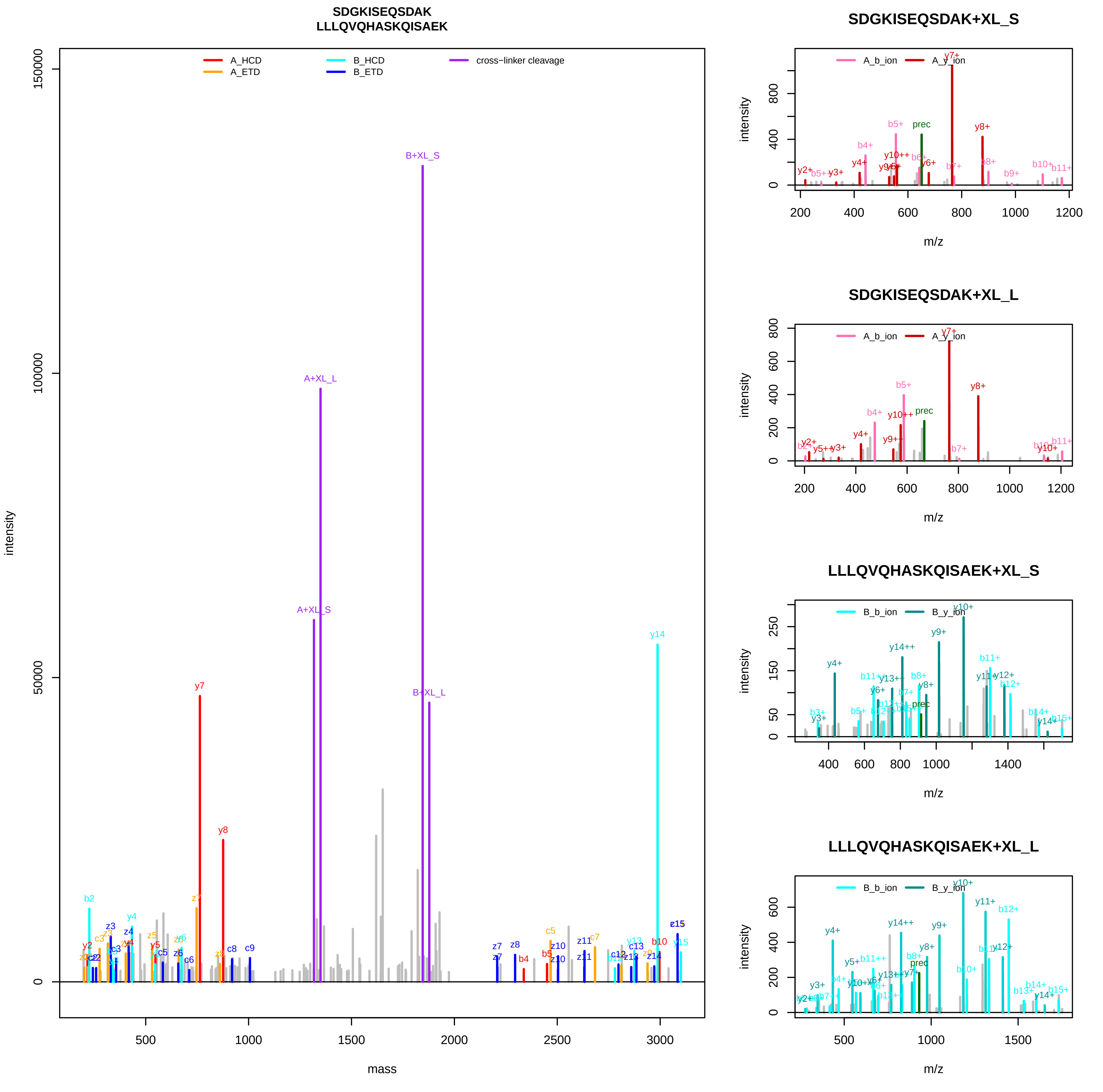

SDGKISEQSDAK
LLLQVQHASKQISAEK
A_HCD
A_ETD
B_HCD
B_ETD
cross−linker cleavage
B+XL_S
A+XL_L
A+XL_S
B+XL_L
y14
y7
y8
b2
y4
z3
c3
y2
y5
z5
z7
c5
c6
z6
c8
c9
z7
z8
b4
b5
c5
z10
z11
c7
c12
c13
y13
z9
z14
b10
c15
z15
y15
150000
100000
50000
0
500
1000
1500
2000
2500
3000
intensity
mass
SDGKISEQSDAK+XL_S
SDGKISEQSDAK+XL_L
LLLQVQHASKQISAEK+XL_S
LLLQVQHASKQISAEK+XL_L
A_b_ion
A_y_ion
B_b_ion
B_y_ion
prec
m/z
intensity
